# Growth and Bone Development in the Horse: When Is a Horse Skeletally Mature?

**DOI:** 10.3390/ani11123402

**Published:** 2021-11-29

**Authors:** Chris W. Rogers, Erica K. Gee, Keren E. Dittmer

**Affiliations:** 1 School of Veterinary Science, Massey University, Private Bag 11-222, Palmerston North 4442, New Zealand; E.K.Gee@massey.ac.nz (E.K.G.); K.E.Dittmer@massey.ac.nz (K.E.D.); 2 School of Agriculture and Environment, Massey University, Private Bag 11-222, Palmerston North 4442, New Zealand

**Keywords:** horse, maturity, physis, epiphyseal cartilage, racing, show jumping, sport, foal

## Abstract

**Simple Summary:**

A comparison of the pattern of growth in the horse with definitions used to describe growth and development in humans demonstrates the same general pattern of growth. In the horse, these development periods are completed very early in life, generally by 2 years of age. Using a variety of measures to define the completion of growth and bone development, the horse enters skeletal maturity by the time it is 2 years old. There is little variation in the age of maturity across different horse breeds. These data support the hypothesis that the horse evolved to be a precocious cursorial grazer and is capable of athletic activity, and used in sport, relatively early in life.

**Abstract:**

Within the lay literature, and social media in particular, there is often debate about the age at which a horse should be started and introduced to racing or sport. To optimize the welfare and longevity of horses in racing and sport, it is important to match exercise with musculoskeletal development and the ability of the musculoskeletal system to respond to loading. The justification for not exercising horses at a certain age is often in contrast to the scientific literature and framed, with incorrect generalizations, with human growth. This review provides a relative comparison of the growth and development of the horse to the descriptors used to define growth and development in humans. Measures of physeal closure and somatic growth demonstrate that the horse completes the equivalent of rapid infant growth by weaning (4–6 months old). At approximately 11 months old, the horse completes the equivalent of the childhood phase of growth and enters puberty. At 2 years old, the horse has achieved most measures of maturity used within the human literature, including the plateauing of vertical height, closure of growth plates, and adult ratios of back length:wither height and limb length:wither height. These data support the hypothesis that the horse evolved to be a precocious cursorial grazer and is capable of athletic activity, and use in sport, relatively early in life.

## 1. Introduction

Within the lay literature, there is often discussion as to the appropriate age, or stage of maturity, to introduce the horse to ridden work. Debate on this topic often appears to be reinforced by statements generated by those opposed to Thoroughbred horse racing and the racing of two-year-old horses [1]. The interesting aspect to this debate is that there is often little consideration, or reference to, either the intervention or epidemiological studies demonstrating the positive effects early exercise has on the reduction in injury and development of the musculoskeletal system [2,3,4,5,6].

Growth in mammals follows a similar pattern to a sigmoid curve, and this is also true for growth in the horse [7]. This pattern of growth, while consistent across mammalian species, does differ in the relative time (age) of the different growth phases as well as the process of the maturation of the musculoskeletal system, both of which are heavily influenced by the ecological niche in which the species has evolved to exploit. Within human literature, growth is often described in three broad stages or categories: a rapid infantile growth, a slower period of steady growth during the childhood phase, and then puberty, with the post pubertal growth spurt followed by the slowing of growth until maturity [8].

The horse evolved as an herbivore to exploit an open plain/grassland environment. Based on the ecological niche, physical and behavioural characteristics, the horse is classified as a cursorial ungulate herbivore. As such, the horse has evolved to escape predation via flight from an early age. Foals are born at approximately 10% of the mature weight and have a musculoskeletal system that is advanced enough to permit standing and suckling within the first hour of birth. Within their first week, the foal is able to regularly cover distances of over 7 kms/day with its dam [9]. A recent review has demonstrated that the horses’ musculoskeletal system, and the long bones of the limbs in particular, are highly receptive to respond positively to loading provided by locomotor play [2]. These data indicate that not only is the foal born with an advanced musculoskeletal system, but that the developmental potential of the musculoskeletal system is highly receptive to exercise, even pre-weaning.

Much of the debate on when to start riding or training a horse is based on the issue of skeletal maturity and the timing of physeal closure either in the appendicular skeleton (long bones of the limbs) or the axial skeleton. To have an informed debate over the age or stage of maturity, at which training should be introduced, it is important to describe the growth of the horse in relation to known patterns of mammalian growth and the relative definition of when a horse is mature.

This manuscript describes growth and bone development in the horse within the framework used within the medical/human literature. The objective of this framework is to permit objective comparisons in order to demonstrate the effect evolutionary programming has had on the rate of bone growth and development in the horse.

## 2. Materials and Methods

Seeding references were obtaining via a structured search within Google Scholar and Web of Science core collection using the keywords horse or equine, and bone and growth, ADG, bodyweight and height. From these references, appropriate references and citations were examined for content. Eligibility for inclusion of studies cited was restricted to the peer-reviewed literature. Estimates for the weighted average range for growth plate closure were based on seeding data from Strand et al. [10] and peer-reviewed published data obtained using the parameters described above.

## 3. Bone Formation

In the developing embryo, bone may be formed by two different processes: intramembranous ossification and endochondral ossification [11]. The majority of the cranial and face bones, and part of the mandible, develop by intramembranous ossification. In this process, mesenchymal progenitor cells form condensations at the future site of the skull bone and differentiate into osteoblasts, which start producing spicules of woven bone [12].

The axial and appendicular skeleton develop by endochondral ossification. Cells from the lateral plate mesoderm (which form the appendicular skeleton) and cells from the paraxial mesoderm (which form the axial skeleton) form condensations of mesenchymal progenitor cells that differentiate into a cartilage template at the location of the future bone [11]. Capillaries, chondro/osteoclasts, and preosteoblasts invade the cartilage, allowing the development of the primary ossification centre. Osteoblasts produce bone osteoids on the scaffold of mineralized cartilage. At the same time, the perichondrium changes to periosteum and a collar of intramembranous bone is formed at the diaphysis. Chondrocytes at either end of the bone continue to proliferate, allowing the cartilage model to grow in length and width. At a certain stage of development, a secondary ossification centre develops at the ends of the long bones, forming the epiphysis. The primary ossification centre, therefore, results in the diaphysis and metaphysis of the long bone, while the epiphysis and its articular cartilage is separated from the metaphysis by the physis (epiphyseal cartilage) or growth plate [12].

The longitudinal growth of bone continues at the physis due to an increase in the number of chondrocytes in the proliferative zone of the physis and an increase in the size of chondrocytes in the hypertrophic zone of the physis. Hypertrophic chondrocytes undergo apoptosis, leaving the remnants of the cartilage matrix to form a scaffold on which osteoblasts form bone osteoids in the metaphysis. The cortex is formed by a combination of bone from endochondral ossification and bone formed under the periosteum from intramembranous ossification [12].

Modelling of bone allows the formation of the characteristic shape of each bone. In a carefully synchronised process influenced by genetic and mechanical forces, bone is removed by osteoclasts from the endosteal surface of the bone cortex and formed by osteoblasts on the periosteal surface; thus, allowing the expansion of the medullary cavity and an increase in the width of the bone [12]. This process is known as appositional growth.

The axial skeleton develops from the embryologic notochord, and somites that differentiate into the sclerotome [13]. The notochord forms the nucleus pulposus of the intervertebral disc, while the other structures of the spine, including the annulus fibrosus of the intervertebral disc, the vertebral body, ligaments, and tendons, develop from the sclerotome [13]. Each vertebral body (secondary mesmerisation) is composed of the caudal segment of one sclerotome and the cranial segment of the adjacent sclerotome [14]. As with the long bones, a cartilage model of the vertebral body is formed [13], and each vertebral body is composed of three primary ossification centres, one for the centre (which makes up most of the vertebral body and is known as the centrum) and one for each vertebral/neural arch [14]. In humans, the C3–L5 (3rd cervical–5th lumbar) vertebrae have five secondary ossification centres that develop at puberty [15]. These occur at the tip of the spinous process, each transverse process, and an annular epiphysis (epiphyseal ring, annular ring) at the upper (dorsal) and lower (ventral) surfaces of the vertebral bodies [14].

The vertebral endplate in quadrupeds and humans differs in structure. In animals, the vertebral endplate consists of cartilaginous and bony parts separated from the vertebral body by a growth plate [16]. The cartilaginous part extends to the disc. In humans, however, the cartilaginous portion extends to the epiphyseal ring, and the growth plate does not exist. Therefore, the longitudinal growth of human vertebrae occurs from the vertebral epiphyseal cartilage [17]. The epiphyseal ring is an apophysis and so does not contribute to longitudinal growth.

## 4. Growth and Bone Development in Humans

### 4.1. Definition of Maturity

Within the human literature, there is still debate about the definition of maturity and the definition of the cessation of bone growth. Bone is highly responsive to external stimuli and loading, and this concept has been described by the mechanostat theorem proposed by Frost [18] and continually refined in the Utah paradigm of skeletal physiology [19]. This theorem proposes that bone responds, in either an anabolic or catabolic manner, to ensure bone strain is maintained within an optimal range. Increases in muscle mass increase bone strain by muscle forces and a subsequent increased ground reaction force and loading strain during limb impact. Thus, bone, dependent on location, will still have appositional growth even once longitudinal growth has ceased [20].

Within the human literature, a threshold for growth velocity is often used as the most pragmatic definition for the cessation of growth. This approach provides a mechanism to assess that the final height has been achieved without the requirement to know the final expected mature height. Within the literature, the range for cessation of growth is between <2 cm and 0.25 cm/year, with most authors converging around ≤1 cm/year [21].

### 4.2. Pattern of Growth

Increases in height follow the sigmoid growth curve with the initial rapid but slowing infantile phase lasting from birth to approximately 3 years old. After this age, until the onset of puberty (the age of which differs between males and females), is the childhood growth phase, where there is a steady increase in height. During the first two phases of growth, the infantile and childhood phase, there are rapid increases in height due to the longitudinal growth of long bones. The longitudinal growth of the long bones contributes more to the increases in height than growth in the axial skeleton, specifically, the vertebrae. During the pubertal growth spurt, there is a cessation of the longitudinal growth in the long bones and increases in the height of the vertebral bodies (see later section on sitting height vs. standing height). Within the human literature, there are documented ethnicity and socioeconomic-related differences in measurements of proportion during growth and at maturity, which may in part be due to differences in genetics, but also cultural influences, often nutritional-related, which alter the rate of linear growth in childhood [22].

### 4.3. Bone Age

Bone age is the methodology to quantify the cessation of bone growth, at the secondary ossification centres (the articular epiphyseal complex) within long and short (cubodial) bones. In humans, the two most commonly used methods rely on radiographing the bones in the hand and the wrist to assess if ossification has occurred and growth has ceased [23].

### 4.4. Peak Bone Mass in Humans

Peak bone mass is the maximal bone content (bone tissue) achieved by an individual, which is usually achieved post adolescence, by approximately 18 years of age [20]. Post-adolescence longitudinal growth in the vertebrae and the long bones (limbs) ceases, but appositional growth (increases in diameter—cross sectional area) continues at a moderate rate for a few years until peak bone mass is attained. The age of attainment of peak bone mass is accelerated by a relative growth rate and the age of the onset of puberty; thus, females achieve peak bone mass earlier than males. Peak bone mass is moderated by exogenous factors such as exercise and muscle mass. Peak bone mass represents a balance between bone resorption and bone formation. With aging (age-related bone atrophy), this balance shifts, resulting in a net loss of bone [8].

### 4.5. Chronological Age

Chronological age is rarely used within the human literature due to the availability of other more precise measures of bone growth and development that are often not readily available for livestock. In the human literature, many studies have used 18 years as the accepted time at which growth has ceased. However, given the inherent variation within a population, estimates of chronological age based on measures of bone maturity (cessation of bone growth) are often presented with a precision of ±2 years [24].

### 4.6. Height and Sitting Height

At the completion of the infantile growth period, when 3 years old, the human infant will be approximately 50% of the mature height. The majority of the increase in height (66%) during this period is due to longitudinal growth in the appendicular skeleton (limbs) [25].

In humans, the relative height to sitting height ratio demonstrates that, at birth, the sitting height represents approximately 68% of height. At 3 years (the completion of the infant growth phase), the sitting height accounts for 57% of height, and at around the time of puberty, this decreases to 52% [25]. These data indicate that, in humans, during the pre-pubertal years, much of the increase in height is due to the growth of the limbs rather than the trunk. Puberty and the post pubertal growth spurt result in an increase in growth velocity with a very subtle increase in the sitting height to height ratio, returning, approximately, to the pre pubertal ratio by 21 years of age [25].

## 5. Growth and Bone Development in the Horse

### 5.1. Bodyweight

**Figure 1:**
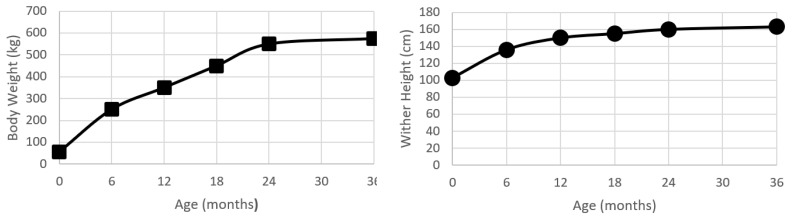
Plots of bodyweight (kg) and wither height (cm) from birth to 24 months age for a population of Kentucky Thoroughbreds. Data modified from Huntington, Brown-Douglas and Pagan [26].

Liveweight, or bodyweight, has been the most commonly used method to describe growth and development within the horse. Recently, Huntington, et al. [26] conducted a review of growth and development in the Thoroughbred. At birth, the Thoroughbred foal is approximately 10% of the mature weight (55 kg), and by the time of weaning (6 months of age) will have had over a four-fold increase in bodyweight to be 43% of mature weight. As a yearling, the Thoroughbred is 61% of the mature weight, and as a two-year old will be approximately 96% of the mature weight [26] (Figure 1). There are some reported regional (country) differences in the growth rate and final bodyweight [26], but the pattern of growth is relatively similar, displaying the typical mammalian sigmoid growth curve [7]. In comparison to humans, horses are seasonal ‘long day’ breeders, and it appears that the attainment of 60% of mature weight in association with the appropriate photoperiod cues permits the onset of puberty, and, thus, growth in horses does have a seasonal moderator on the growth curve [27].

The published literature on bodyweight for other breeds of horses is relatively limited, and data were often collected to support the primary aims of the studies. In the Dutch warmblood, the foal demonstrated the same four-fold increase in weight by the time of weaning (5–6 months old) and were ~62% of expected mature weight at weaning [28]. In the Selle Francais and French Trotter, the pattern of growth for bodyweight and wither height was the same as for Thoroughbreds [29]. In a study examining the effect of dam size on the longitudinal growth and osteoarticular status, the pattern of growth was consistent even with dramatic differences in dam size due to breed [30]. The similar patterns of growth reflect that birthweight (which is influenced by dam size and intra-utero growth) accounts for much of the variation in the absolute growth rate, but that the pattern of growth is heavily conserved across equine breeds.

### 5.2. Wither Height and Body Length

The wither height has a sigmoid growth curve that is similar in shape to the increases in bodyweight (Figure 1). At birth, a foal is approximately 60% of the mature height. By 24 months of age, the foal will have almost doubled in height, with the majority of this growth being achieved by longitudinal growth in the proximal limbs and the thorax.

#### 5.2.1. Thoroughbred

The majority of data on growth in the Thoroughbred has been collected using bodyweight, with relatively less data recorded for wither height. This may be due to the relatively greater ease in which bodyweight data can be collected with young horses, rather than other measures such as wither height. At birth, Thoroughbreds have a wither height of 106–109 cm [26] and achieve approximately 98% of the mature height at 24 months of age. The wither height has a relatively high heritability. Periods of nutritional deficit may result in a temporary inhibition of wither height growth trajectory, and while this may delay the attainment of the final height, it generally does not prevent the attainment of the expected wither height [31].

Despite the Thoroughbred often being touted as an early maturing breed compared to other horse breeds, the pattern of growth and attainment of a near final wither height is very similar across all horse breeds [10,30], even within heavy draft crosses bred for meat production [32]. The pattern of growth between cohorts of Thoroughbred, Selle Francais, and French Trotter horses showed a remarkable similarity, with a near flattening of the growth curve reported at 2 years of age [29]. A similar agreement in wither height growth curves between Thoroughbred, warmblood, and crossbred horses was modelled by McManus, et al. [33]. In the Lipizzaner, mature height was estimated to have been achieved slightly later by 27 months of age [34].

#### 5.2.2. Relative Growth—Back Length and Sitting Height

In the horse, growth in the back length is as close an analogue to the sitting height and sitting height growth velocity in human studies. The comparisons between sitting height and back length are entirely conceptual, given human are bipedal and horses are quadrupedal, but the limited published data do indicate that, in the horse, growth in the wither height does provide a relatively robust proxy for growth in the spine [35]. In sheep, which are often used as an animal model for human orthopaedic research, most growth in vertebra length occurs between 3 and 6 months of age, which is analogous to the human childhood growth period between 4 years and puberty [36]. Morphometric data from young Thoroughbreds indicate that at birth, the back length to wither height ratio is approximately 0.8:1, at 12 months it is 0.93:1, and by 18 months of age is 0.99:1 [37]. This data, in association with the reported closure times of the different vertebral physes [38], indicate that the relative longitudinal growth in the spine ceases around the time of cessation in the wither height growth.

### 5.3. Bone Growth and Development

**Figure 2:**
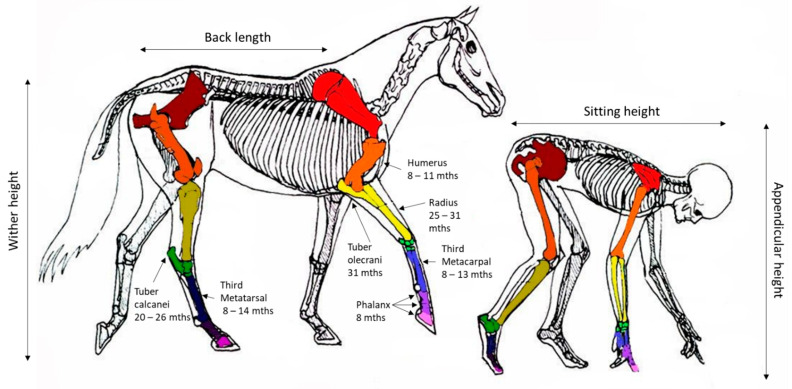
Representation of the equivalent anatomy of human and equines with the reported age (months) at closure of specific growth plates (physis) for the horse based on weighted average estimates presented in Table 1. Image modified from *Centered Riding* by Sally Swift.

Changes in the wither height and the timing of cessation in growth provides the best indicator of bone growth velocity. In the horse, the majority of the increases in the wither height are achieved by growth in the thorax and, to a lesser extent, the proximal limb. The requirement for early locomotion appears to have resulted in the bones in the distal limb at birth having a structure and size similar to those at maturity and, therefore, having a relatively limited capacity for longitudinal growth. Figure 2 provides a comparative image of the skeleton of the horse and human and the relative estimates of growth plate (physis) closure in the respective major bones of the limb based on the weight average estimates presented in Table 1.

Similar to humans, there is some expectation that the peak bone mass, due to subsequent appositional growth, may occur after longitudinal growth ceases. In humans, DXA (dual-energy X-ray absorptiometry) of the lumbar spine is routinely used to quantify bone mass and much of the literature on peak bone mass utilises data captured using this technique. DXA is relatively easy to use and cost-effective in humans, but this is not the case when quantifying bone mass in horses. At present, there is a lack of comparable equine data, particularly in relation to the axial skeleton, to definitively identify when peak bone mass is achieved within the horse. The most robust data unfortunately focused on the distal limb, rather than the lumbar spine, in race-trained Thoroughbreds that had been conventionally reared or exposed to preconditioning exercise [5]. These data demonstrated no increase in bone circumference (periosteal circumference) in the proximal phalanx, but the training programme induced an increase in the periosteal circumference within the third metacarpal bone [39]. At present, there are limited data describing appositional growth in the vertebrae of the horse. An indirect finding of a study developing a cervical disc index for horses showed there were limited changes in the size or morphology of cervical vertebrae after 18 months of age [35]. These data indicate that, in the cervical spine of the horse after 18 months old, when there is an associated attainment of a near-final wither height, there is limited growth in the horses’ vertebrae.

Longitudinal growth originates from the physis (growth plate), which, during periods of growth, can be observed either via radiographs or histologically as hyaline cartilage. Once longitudinal growth has been completed, the physis ossifies and the radiolucent line of the physis is no longer radiographically visible. Radiographic surveys have been utilised to quantify the age at which there is a cessation of longitudinal growth. However, as identified by Fretz, et al. [40], the cessation of longitudinal bone growth precedes physeal closure. This effectively means that the relative age at which physeal growth ceases may be a number of months earlier than that identified based on radiographic studies.

Across a variety of breeds, the pattern of growth plate closure is consistent, even in breeds considered to be slow maturing. Table 1 was adapted from Strand, Braathen, Hellsten, Huse-Olsen and Bjornsdottir [10], and summarises the ages in months for the radiographically reported closure of growth plates in horses’ limbs. For the reported studies, a weighted average for the range of growth plate closure was estimated. An interesting observation is the relatively tight ranges presented across studies despite differences between breeds and small sample sizes.

## 6. Overlaying Human Principles of Growth with the Horse

**Figure 3:**
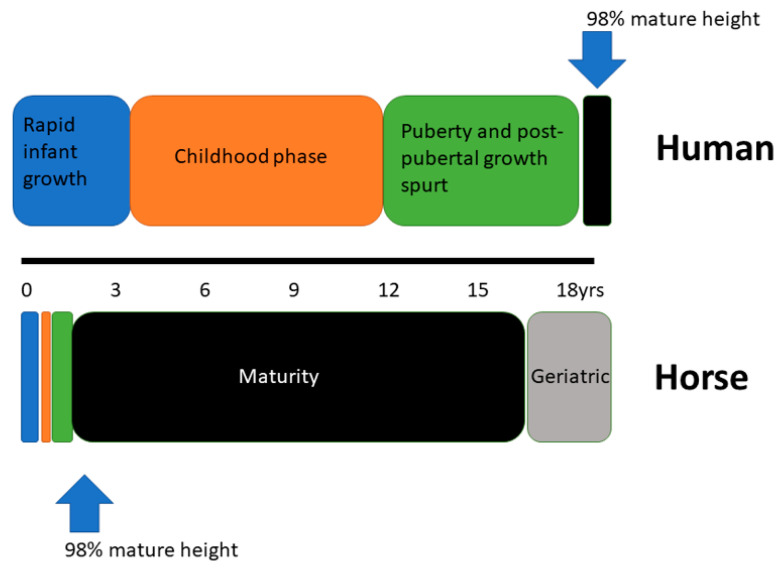
Schematic representation of the three periods of growth in human and equine development and the relative age at attainment of skeletal maturity.

In contrast to human growth and development, the horse is born as a precocious cursorial species. The requirement to avoid predation and be capable of covering significant distances while the dam grazes has altered the relative temporal pattern of the growth phases used to describe human growth. Figure 3 provides a graphical representation of the relative contraction of the different periods of growth in the horse and the early attainment of maturity relative to humans.

In the horse, the equivalent of the rapid infantile growth period lasts from birth to weaning (4–6 months of age). Published data for the growth in wither height and increases in body weight support this, with the inflection point in growth consistently occurring at weaning, irrespective of the production system or breed of horse. It is during this period that there is the most rapid longitudinal growth in the horses’ distal limbs and increases in bodyweight, with a rapid development of muscle mass. Based on data presented for Thoroughbred foals at the start of this period of growth, the limb length (appendicular) contributes 68% of the wither height, and this relative proportion reduces to 63% by 6 months of age [37]. This reduction in the relative contribution of the limb length to wither height demonstrates the relatively greater growth observed in the proximal limb and thorax/axial skeleton rather than the appendicular skeleton of the horse.

The second phase of human growth is the childhood phase, which is characterised by a steadily decreasing growth rate up until the onset of puberty. During this growth period in humans, there is moderate growth in both the axial and appendicular skeleton. The decreasing sitting height to height ratio indicates that, during this phase, there is a more rapid rate of growth in the appendicular skeleton than the axial skeleton [25].

In horses, the childhood phase is equivalent to the period post-weaning (4–6 months) to 10–11 months of age. During this period, there is a reduction in the growth rate with ADG (Average Daily Gain) reducing to approximately 0.6 kg/d. The ADG decreases consistently until a nadir at approximately 10–11 months of age. At this age, most horses will have achieved the minimum bodyweight for puberty and the seasonal environmental cues (photoperiod) are sufficient for the expression of puberty [27]. In the southern hemisphere, where horses are kept at pasture, this equivalent to the childhood period with the nadir in ADG coincides with winter. However, the same pattern of growth is also observed in horses intensively managed, which supports the hypothesis that this reduction in the relative growth rate is driven by developmental biology rather than environmental cues and changes in pasture quality and quantity. At the completion of this growth phase, most of the growth plates distal to the carpus and hock have radiographic evidence of ossification (Table 1). These data, in association with the observation that longitudinal growth ceases several months before radiographic evidence of ossification, indicates that the distal limb contributes little to increases in either the leg length or wither height (Table 2). Increases in wither height during this period of growth occur in the proximal limb and axial skeleton, with the relative contribution to growth being equally divided between the axial skeleton and the proximal limb.

The third phase of human growth is the adolescent growth spurt that is marked by an initial period of rapidly accelerating growth velocity, followed by a deceleration until the final adult height is reached. There is significant variation in the age that puberty is attained and the start of the respective growth spurt. Much of the variation in the age of puberty can be explained by the relative proportion of adult bodyweight attained, as the onset of puberty is heavily regulated by bodyweight in mammals. Puberty in horses can occur from as early as 8 months of age, but typically occurs at 10–11 months. The largest datasets on horse growth belong to the Thoroughbred, and the effect of the production system may influence the presentation of growth around the time of puberty as this coincides with spring and an increasing focus on the production of well-grown yearlings for the yearling sales. Despite this possible management confounder, the tight seasonal grouping of the ADG nadir in August (in the southern hemisphere), irrespective of birth month [26], in association with the onset of puberty data [27], indicates that there is a probable pubertal growth spurt in the horse. At the onset of puberty, horses are approximately 92% of the mature height and 60% of the mature weight. It has been reported that, in healthy humans, this pubertal growth spurt occurs at 90% of the final height [8], which is similar to that observed with horses.

In humans post puberty, there is a relative reduction in the longitudinal growth of the appendicular skeleton (limb length), but a subtle increase in the sitting height, indicating continued bone apposition in the vertebrae. This trend is observed in horses with 98% of the mature height being attained by 24 months of age and a very moderate/subtle apposition of bone occurring in the axial skeleton. This moderate acquisition of bone in the axial skeleton is probably in response to the subtle lag between the final wither height and final body weight.

Due to the inability to use DXA scanning to quantify peak bone mass in horses, the closest proxy to quantify bone turnover is the use of serum markers of bone activity. However, the association of serum bone biomarkers and skeletal growth can be problematic, particularly when measured in isolation. This is because markers of bone formation, such as bone alkaline phosphatase (bALP) or osteocalcin, cannot discriminate between longitudinal growth, and appositional growth or bone modelling. Generally, in humans, bone biomarkers peak during the growth spurt at puberty and then decline to adult concentrations [52]. However, the association of these biomarkers with growth or bone mass in the human literature can be conflicting. For example, some studies have shown an association between bone biomarkers and height, but the biomarkers were not predictive of bone gain (bone mineral content and density) [53,54]. Conversely, other studies have shown that there are a number of determinants of serum biomarker concentration, including the sexual maturity rating (Tanner stage), sex, height, velocity, and whole-body bone mineral content [52]. This study found that, for children with the same sex and sexual maturity stage, and with similar growth velocities, children with a greater bone mass and bone mineral content (BMC) had greater biomarker concentrations. Additionally, the genotype of an individual may be a complicating factor [52]. For example, in one study, the ability of serum bone biomarkers (total ALP, bALP, type I carboxyterminal telopeptide) to predict the bone mass, height, and sitting height increase depended on the vitamin D receptor (VDR) *Fok1* genotype of the individual [55].

In Icelandic horses, the total Alkaline Phosphatase (ALP) activity (both the bone fraction and liver fraction) decreased rapidly during the first year of life, and has appeared to plateau at 24 months of age [10]. This plateau in ALP reflects the near cessation of growth in the wither height and back length. However, the levels from 24 months to 60 months did not decrease to the level of a control group aged 7–16 years. The 60-month-old horses were in light work and the effect of loading and increased muscular strain on appendicular and axial skeleton may in part explain why serum ALP activity was above that reported for the aged control group. These data indicates that, similar to humans post-puberty, there is ongoing appositional bone growth in response to load, but according to most definitions of skeletal development, maturity in the horse is achieved by 24 months old.

## 7. Conclusions

Both horses and humans demonstrate similar patterns of growth, but in the horse, these phases of growth are heavily restricted to the first two years of life. This condensing of the growth phases and early attainment of maturity reflects the ecological niche to which the horse evolved to exploit as a cursorial herbivore. An examination of the literature on measures of bone growth and maturity supports the hypothesis that the majority of growth is completed before the horse is two years old. These data, in combination with controlled experimental studies and epidemiological studies examining large complex datasets on training and exercising young horses, reflect the hypothesis that evolutionary programming means the horse is capable of positive musculoskeletal responses to exercise early in life. Current industry practices of racing and training 2-year-old horses are in alignment with the horses’ developmental potential and evolutionary programming.

## Figures and Tables

**Table 1:** Radiographically reported closure of growth plates in the horses limb and weighted average estimate of age (months) of physeal closure for anatomical sites in the limb: modified from [10].

Breed	Reference	*n*	Proximal Second Phalanx	Proximal First Phalanx	Distal Third Metacarpal	Distal Third Metatarsal	Distal Radius	Proximal Radius	Tuber Olecrani	Distal humerus	Tuber Calcanei
Brazilian Thoroughbred	[41]	20					20.9–27.6				
Thoroughbred	[42]	800		8.0–14.0	8.0–14.0						
Thoroughbred	[43]	53									16.0–24.0
Thoroughbred	[44]	19					20.7–33.1				
Thoroughbred–Quarter Horse Cross [11]	[40]	9		6.0–10.0	7.0–9.5	9.0–12.5	24.0–25.5				
American Standardbred	[45]	113					26.0–35.0				
Standardbred	[46]	14					24.2–31.9				
American and Italian Standardbred	[47]	140					26.0–29.0				23.0–27.0
Standardbred	[48]	33					26.0–33.0				19.0–28.0
Arabian [14]	[49]	2	7.5–7.9	7.5–8.8	7.0–7.5	7.0–7.5	23.2–23.7	13.6–14.0	26.6–29.7	13.6–14.9	
Arabian	[44]	20					19.8–27.0				
Anglo Arab	[44]	21					24.8–29.7				
Hucul	[44]	21					22.1–30.2				
Brazilian Manga-larga [17]	[50]	7					24.6				
Finnhorse	[51]	15					24.0–30.0				
Icelandic horse	[10]	35–56	8.1	8.1–8.5	8.1–8.5	8.1–14.9	27.4–32.0	14.9	31.5–32.2	8.8–11.0	19.0–26.7
Calculated weighted average range (months)			8.08–8.09	7.9–13.6	8.0–13.6	8.2–14.4	25.1–31.1	15.15–15.16	31.3–32.1	9.0–11.1	20.6–26.3

**Table 2:** Summary of the growth phases used to describe human growth and the relative timing of these in horses with the criteria used to define attainment of these growth or maturity stages.

Pre Utero Growth	Birth	Rapid, Decelerating Infantile Growth Phase	Childhood Phase	Puberty	Post Pubertal Adolescent Growth spurt	Onset Maturity
**In humans (age timeline)**		Infantile to 3 years old	3 yrs. to puberty (around 10 years for females—body weight dependent)	12–14 years (bodyweight and gender dependent)	Puberty to late adolescent	~18 years
**In horses (age timeline)**		Birth to 4–6 months old	Weaning to long weaning (6 months–10 months)	Primary ~60% mature body weight and photoperiod (10–11 months)	Yearling to long yearling (12–18 months)	~24 months
**Relative weight** **(% mature value)**	10%	10–43%	43–61%	61%	61–78%	96% mature weight
**Relative height** **(% mature value)**	63%	63–83%	83–92%	92%	92–95%	98% mature height
**Bone age (skeletal maturity)**						Distal radius 25.7–31.1 months
**Peak bone mass**						Based on ALP levels inflection at 24 months
**Ratio: back length: wither height**	0.78:1		0.93:1	0.96:1		1:1
**Ratio: appendicular limbs: wither height**	0.67:1		0.62:1	0.60:1	0.60:1	0.60:1

## Data Availability

All data are presented in this study. Original data may be found within the respective manuscripts cited.
